# “*Sometimes it feels like thinking in syrup*” – the experience of losing sense of self in those with young onset dementia

**DOI:** 10.1080/17482631.2020.1734277

**Published:** 2020-02-28

**Authors:** Laila Mohrsen Busted, Dorthe S Nielsen, Regner Birkelund

**Affiliations:** a Department of Regional Health Research, Faculty of Health Sciences, University of Southern Denmark, Odense, Denmark; b Health Sciences Research Center, UCL University College, Odense, Denmark; c Migrant Health Clinic, Odense University Hospital, Center for Global Health, University of Southern Denmark, Odense, Denmark; d IRS, Lillebaelt Hospital, University Hospital of Southern Denmark, Denmark

**Keywords:** Young onset dementia, qualitative research, thematic analysis, reflexive, semi-structured interview

## Abstract

**Purpose**: To explore and describe the experience of people having young-onset dementia.

**Methods**: This was a qualitative study that used semi-structured interviews to collect data from nine persons with young-onset dementia (aged 47–65; five men and four women). Data were collected in the spring of 2018. All interviews were conducted at the participants’ choice and in their own homes by one interviewer. The collected data were analysed using the six-stage process of reflexive thematic analysis model.

**Results**: The analysis revealed three themes: Dementia causing loss of control over oneself; becoming a burden to the family while sense of self disappears; and fearing a humiliating future.

**Conclusions**: The experience of having and living with young onset dementia affected the persons’ thoughts and memory and was experienced through the persons’ loss of personality and sense of self. Thoughts about the future were associated with fear, and the risk of changing their personalities to something different from the one which they had experienced as humiliating throughout most of their lives.

## Introduction

The experience of encountering dementia has been described by people with dementia as loss of control, loss of role and loss of identity (Clemerson, Walsh, & Isaac, 2014; Spreadbury & Kipps, 2018). Being diagnosed with young onset dementia is considered to be a disruption of the life cycle since it is unexpected and out of time with their biography both to them and those who know them (Clemerson et al., 2014; Greenwood & Smith, 2016). It is a rare condition and is also less common than dementia, which comes in later stages of one’s life (Prince et al., 2015; Vieira, 2013). Young-onset dementia is defined as dementia with symptom onset before the age of 65 (Draper & Withall, 2016). Recently, there have been discussions in the literature related to the estimation of the number of persons with young-onset dementia (Kvello-Alme, Bråthen, White, & Sando, 2019). However, it is estimated that about 2300 people are living with young onset dementia in Denmark (Jørgensen, 2019).

The management of young-onset dementia presents different challenges from those found in dementia among older persons, mainly because they usually still work when their symptoms emerge, thereby incurring in more financial hinderances (Greenwood & Smith, 2016). Therefore, due to the nature of the condition, changes in job performance or behaviour experienced by those with young onset dementia are not always understood by other people in their surroundings (Clemerson et al., 2014; Evans, 2019). Moreover, it has been shown that people with young onset dementia are often parents of young adults or teenagers, so they usually have family responsibilities (Rossor, Fox, Mummery, Schott, & Warren, 2010), and some also still have older and healthy parents owing to their young age.

In terms of the impacts of dementia, it has been shown that there are implications for one’s sense of self-confidence and that it is strongly associated with disempowerment. A previous study has shown that living with the disease involves feelings of uncertainty and becomes a struggle between self-protection and self-adjustment (Steeman, De Casterlé, Godderis, & Grypdonck, 2006). The condition creates a need to maintain a sense of being useful for one’s family and surroundings, and this happens especially when the condition requires the person to cease working. This poses an enhanced importance on the maintenance of purposeful activities in the early stages of the condition (Roach & Drummond, 2014; Van Vliet et al., 2017). Further, the uncertainty about how the disease may develop seems to cause devastating psychosocial consequences, and it is well known that the whole family experiences a profound sense of loss when the person is diagnosed with young onset dementia (Cabote, Bramble, & McCann, 2015). It occurs not only owing to dementia symptoms but also owing to subsequent changes to lifestyle and roles (Svanberg, Spector, & Stott, 2011).

Family members feel like they are being “robbed of their future”, and there is also guilt associated with having these feelings towards the person diagnosed with young onset dementia (Svanberg et al., 2011). Having to manage role changes and becoming “like a parent” for the person with dementia seem to change their feelings towards those diagnosed. For instance, spouses tend to experience a gradual protective behaviour, so they end up demonstrating more rigid control towards the person with dementia, who thereby may be at risk of feeling controlled and being treated like a child (Wawrziczny, Antoine, Ducharme, Kergoat, & Pasquier, 2016). In corroboration, previous studies have shown that people with dementia experience a struggle for autonomy in their lives and an increasing dependency on others (Clemerson et al., 2014; Johannessen & Möller, 2013; Spreadbury & Kipps, 2019).

Based on the previous studies, we deem that there is a need for further knowledge regarding the experience of living with young onset dementia (Clemerson et al., 2014; Spreadbury & Kipps, 2019). Qualitative research, with its ability to provide insights about the subjective experience of lived phenomena, should be well placed to offer answers. Among many stakeholders, this type of knowledge is especially important to health professionals, as it allows them to know how to appropriately shift towards providing person-centred care for these specific types of patients (Kristiansen, Normann, Norberg, Fjelltun, & Skaalvik, 2017; McKeown, Clarke, Ingleton, & Repper, 2010). Thus, this qualitative study aimed to explore and describe the experience of people having young-onset dementia.

## Materials and methods

### Design

This study was conducted as a qualitative study using semi-structured interviews inspired by Kvale and Brinkmann and was conducted to get detailed information related to the topic in examination (Kvale & Brinkmann, 2015). Data were analysed using Braun et al.’s model for reflexive thematic analysis (Braun, Clarke, Hayfield, & Terry, 2018).

### Participants and recruitment

In total, there were nine participants, five men and four women, where eight were diagnosed with Alzheimer’s and one with vascular dementia. See Table I for more details on the description of the participants. They were recruited by the help of dementia consultants who identified participants who were willing to participate and consulted their families regarding this participation. Inclusion criteria included being under 65 when diagnosed with dementia and who were assessed by dementia consultants as being able to give consent to participate, both verbally and in writing. The principles of purposive sampling were adopted to ensure diversity in terms of age, gender, diagnosis, and living arrangements (Moser & Korstjens, 2018). The participants’ ages ranged from 47 to 65 years old, with a mean age of 58 years.

**Table I.** Description of the participants

	Participant, diagnosis, age	Time since diagnosis (months)	Living arrangement
Interview P1	Man, Alzheimer’s, 64 years	8	Living with wife
Interview P2	Woman, Alzheimer’s, 51 years	12	Living with husband
Interview P3	Woman, Alzheimer’s, 52 years	5	Living with partner
Interview P4	Man Alzheimer’s, 61 years	5	Living with wife
Interview P5	Man, Alzheimer’s, 63 years	3	Living with wife
Interview P6	Woman, Alzheimer’s, 62 years	12	Living alone
Interview P7	Man, Alzheimer’s, 63 years	3	Living with wife
Interview P8	Man, Alzheimer’s, 65 years	3	Living with wife
Interview P9	Woman, Vascular dementia, 47 years	15	Living with husband

Further, to be able to participate in the study, participants must have had been able to demonstrate their understanding of it, so the researcher asked the eligible participants to re-articulate the study´s purpose and to describe how they would be able to contribute to it. As a counterpart, some participants wished to have a family member present during the interview to promote a trustful environment and ensure their protection, and this was granted to them.

### Data collection

Data were collected in the spring of 2018. All interviews were conducted at the participants’ choice and in their own homes by one interviewer, who had experience in homecare nursing for people with dementia. Two of the interviews were conducted as individual interviews, and seven interviews were conducted as interviews with partner, so either the patients’ spouse or another family member was present in the room. To ensure the interviews were focused on the person with young onset dementia, the family members were briefed that they should avoid helping the patients when they were answering and were asked not to intervene or finish sentences for the participants. The participants with young-onset dementia were ensured that they would have all the time needed to articulate and narrate their experiences without being interrupted.

In view of the pre-understanding established both by the existing literature about the experience of having young-onset dementia and the interviewer’s (LMB) experience of working with people with dementia. The pre-understanding brought the interview situations knowledge about what to expect from the people with young-onset dementia. Specially on how to communicate and to pose short and easy understanding questions but also offering patience and time for the person with dementia to answer the question during the interview. Based on this knowledge, a set of open-ended questions was developed and was utilized as an interview guide.

As an example, the participants were asked: “Please, tell me: how do you experience dementia in your everyday life?*”* and “What kind of thoughts do you have about your future?*”*. The interviews lasted between 58 and 112 min. All interviews were recorded on a Dictaphone and transcribed verbatim by the interviewer. The transcribed interviews were complemented with written field notes by the interviewer. Written notes included observations of both verbal and non-verbal behaviours as they occurred, and immediate personal reflections about the interview situation (Phillippi & Lauderdale, 2018).

### Data analysis

Reflexive thematic analysis is a qualitative method utilized to identify patterns of meaning across a dataset that provide an answer to the research question (Braun et al., 2018). Braun et al.’s six-stage process of reflexive thematic analysis was used to describe the participants’ experiences related to having young-onset dementia. With an inductive approach, codes and themes were developed from data analysis (Braun et al., 2018).

First, data were read and reread several times. In the second step, initial codes were generated using broad codes such as “irritation to oneself” and “embarrassment”.

In the third step, the intersection of data, researcher experience, and subjectivity with the research question allowed us to construct themes, mould them, and give them meaning. This process added more details to the codes, and the codes were combined to construct themes such as “fear of embarrassment in relation to others” and “embarrassment to oneself”; this step ended in a collection of candidate themes and sub-themes.

In the fourth step, the candidate themes were revised and reviewed to check how each theme related to the other themes and to the entire data set. Then, in the same step, a thematic map of the analysis was made to illustrate participants’ phrasal expressions, and the themes were outlined based on the interpretations of these expressions.

In the fifth step, themes were defined and named by identifying the “essence” of what each theme was about. All three authors took part in thorough discussions that were aimed at identifying and refining themes.

The sixth step was writing-up the final report, revisiting the research question, notes, and codes, all to ensure that the final themes remained close to the original data and answered the research question with accuracy. The first author was responsible for the first, second, and third step of the analysis process and all three authors participated in the final steps of the analysis. Findings were discussed and interpreted in the light of existing research and Buber’s thoughts on I-thou relations as a theoretical perspective in the reflection of some of the findings.

### Ethical considerations

Interviewing persons with dementia requires high moral sensitivity (Heggestad, Nortvedt, & Slettebø, 2013). One of the reasons these specificities are required is due to people with dementia and their family members being in a vulnerable situation because dementia affects many domains of the person’s life, acting as a threat to individuals’ identity, autonomy, and independence (Pesonen, Remes, & Isola, 2011). The interviewer [LMB] had solid knowledge and experience working with people diagnosed with young onset dementia in homecare nursing, which contributed to an attentive and sensitive approach during the interviews. Participants’ wishes and needs for having family members close to them during the interviews were respected.

The participants were introduced to the study both verbally and in writing, and they all gave written consent to participate. The ability to provide informed consent by the person with dementia was assessed by the researcher during initial contact. All participants were assured that their participation was voluntary and that they could withdraw from the study at any time without giving any reason. The participants also received assurances of anonymity and confidentiality.

The Danish Data Protection Agency approved the study in accordance with the Act on Processing of Personal Data No. 2015-57-0016. Ethical clearance was obtained from a Danish Regional Committee on Health Research Ethics (S-20162000-158), and approval was not required according to Danish law. The study was conducted in accordance with the Declaration of Helsinki.

## Results

All participants presented that the overall burden of having young-onset dementia was that the disease affected thoughts and memory, which consequentially and severely impacted their sense of self and personality. This caused possible loss of control and sense of self. Three broad themes emerged from the analysis and were used to describe the recurring topics, which account for participants’ experience of losing their sense of self and their thoughts on the future while having young-onset dementia:

1. Dementia causing loss of control over oneself;
2. Becoming a burden to the family while the sense of self disappears;
3. Fearing a humiliating future.

### Dementia causing loss of control over oneself

#### Feeling embarrassed

The experience of living with young onset dementia varied from day to day among participants. Every morning, when waking up, participants could immediately notice in their minds whether today would be a good or a bad day. Some days they were able to think clearly from the morning, and other days they had the feeling that their brain was thinking slowly. A participant described with a metaphor how the disease affected her, which illustrated how the ability to think was getting difficult with time: “Sometimes it feels like thinking in syrup—it is possible, but it takes a long time, it’s sticky and it’s difficult …” P9, woman. Having young-onset dementia was experienced by participants as being a new process of understanding of oneself, and in this process, they experienced both a frustration with themselves for not being able to do what they wanted and a desire for the situation to come back to as it used to be. In that regard, a participant expressed: “I am not myself anymore. There are so many things I cannot remember, and things are going too fast for me at times”. P3, woman.

Further, most participants expressed it was important that they sustained their personality and stayed as normal (in a reference to the way they had always been prior to the dementia symptoms) as possible. In that topic, a man expressed: “I don’t want to be him-with-dementia. I want to live as normal as possible. I don’t want to see others with dementia. I don’t want to be put into a box” P5, man. This quote illustrates the participant’s need to not let the diagnosis take over the control of his life, his fear of being stigmatized and put aside, and his feelings of personality and sense of self.

Participants used different strategies to cope with everyday life. One good example is one participant: she always used the same parking space when going to town, so that she could find her car again; another participant carried bags with names from the shops he had to go to when he went shopping, so as to remember where he was going. Moreover, participants said that they made what was possible to sustain their own identities and sense of self as long as possible by using strategies that prevented the dementia diagnosis to take over and define who they were.

#### Feeling shame

Participants experienced the fact that they were not always able to remember things as frustrating, and they felt this experience was both a torment and a shame in relation to people close to them. They also expressed that they knew their behaviour was changing, and that they knew sometimes they would repeatedly ask for the same things and/or repeat their actions over and over. When they became aware of this situation, it caused them to feel frustrated. Regarding this frustration, a male participant said: “I know I repeat myself, and when I notice it, I get so annoyed! I try to pretend I don’t notice it because the others don’t need to know that I am aware of it”. P1, man.

The quote indicates that, for this participant, realizing he was performing a repetitive behaviour brought him shame, and that he needed to cover it up to hide his embarrassment. This was a common experience among participants. Participants independently expressed that they were well aware of their dementia disease and that they often chose to be open about the disease when meeting new people, which served as an intentional strategy to minimize the embarrassment and shame that would eventually come over them when others experienced their repetitive and forgetful behaviour that were characteristic of their diagnosis.

During the interviews with a family member present, participants clearly showed nonverbal trust and signs of dependency on the family member by eventually turning their head to the family member in a search for help to find the missing words in the conversation. This behaviour not only symbolized how participants would nonverbally express their need for help to find the right words for the conversation but also that they sought help to not instinctively reveal both the embarrassment and shame of not being able to speak for oneself as usual.

### Becoming a burden to the family while the sense of self disappears

#### Feeling like a burden in the marriage

Participants who had a partner explained that hurting their partner was the worst element about having the disease. They expressed how they would easily forget about having dementia and what this meant to their relatives. When they expressed these situations, it would usually be related to feelings of fault and having a guilty conscience for not being able to help with practical things around the house and not being able to have conversations and intimacy as they used to in their marriage. This made the participants feel being a burden to the family. Participants also expressed awareness that they had to keep a low profile when being around their spouses or partners, as they knew that their repeating behaviour and a repetitive sequence of questions could overburden their spouses or partners, especially so when they recognized the sighing and the encouragement to think twice before asking the same questions that came from the latter. A participant illustrated this by saying: “My husband is very understanding, but he is busy. He has become wrinkled, and he looks devastated. It hurts me to see. I think I take up too much space”. P2, woman.

This feeling of being a burden to the marriage was obvious when participants expressed their limitations: not being able to help with cooking and/or housekeeping anymore, or even not being able to have conversations as they used to have. Further, ceasing to work, apart from the psychological effects, also had financial consequences for the marriage, for family life and towards their routines, and participants also expressed this overall lack of contributions to the family was as a burden. The feeling of being incapable, added to the perceptions that they were the ones who were causing the family’s future and possibilities to be taken away from them, also entailed the feeling of being a burden to the marriage.

#### Feeling like a burden to the family

All participants had children, and most of them expressed that they had a close relationship with their children by speaking openly and freely to each other about their feelings. They reported having frequent contact with each other and, for some participants, they experienced that the relationship felt even better than before the diagnosis. At the same time, participants were aware that the children were broken-hearted by having a parent with dementia, and causing sadness to their children was perceived as extremely burdensome.

Additionally, the relationship with their children was marked by the uncertain future, which in most families, in turn, resulted in a greater confidentiality between the children and the person with young onset dementia. Participants were also well aware that, if something needed to be said, it had to be said now because their memory could disappear in the future. However, not all participants expressed a closer and better relationship with their children; one of the participants stated that her relationship to her 19-year-old son had become more difficult and that he needed more distance from her because of her dementia.

The relations to siblings, parents, and peripheral family members seemed to change as well. In this regard, a participant expressed: “My siblings don’t visit me much anymore, perhaps to protect me. I guess they think I have a busy everyday life”. P2, woman. Another participant expressed it this way: “Unfortunately, my parents bury their heads in the sand. They keep a distance from us [the participant and her partner]*”*. P3, woman. Finally, participants who had troubled relationships with their parents and siblings explained that they were afraid that they had caused their families to become distant, which also contributed to their feelings of being a burden to their families.

### Fearing a humiliating future

#### Fear of forgetting and being forgotten

The fear of forgetting was an issue of major concern to the participants. If it was glasses or the keys, it did not mean much, but the fear of forgetting the important things in life—like family and friends—was devastating. This sentiment was also expressed by their fear of being forgotten, so much so that their children or spouses would eventually forget who they had once been within their relationships, and that this forgotten memory would, thereby, give way for the memory of the person with dementia.

It was obvious to the participants that their changing behaviours would eventually lead to their dependency on others. Based on their data, we found that these two thoughts—turning into a helpless and dependent person and the risk of being forgotten as the person they used to be—were associated. A participant described that she knew she was at risk of changing her own personality, and that she risked being dependent on her husband to help her in her day-to-day life. She feared this could lead him to experience caregiver-burden and make him ill, and this responsibility was unbearable to her.

“In the beginning, I did not want to accept this kind of life. I asked for divorce, but my husband didn’t want it. Nevertheless, I signed the papers, and now the decision is his. I set him free. If I get to be dependent on caregivers, I don’t want him to feel obligated”. P10, woman.

This phrase was an example that the thought of being a burden to her husband was so humiliating that she had convinced him to live in open divorce. She preferred to set her husband free and be dependent on other people instead of him. This agreement had given her peace about the future, knowing that she would have avoided the humiliation of being a burden to her beloved husband and that she gave him the possibility to leave her with blessings when the dementia became severe enough, so that he remembers her as the person she used to be.

It was also characteristic that all participants expressed that they took one day at a time and made sure they had a good day every day. Further, most participants found it hard to acknowledge and to talk about the progression of the dementia with their families. For the participants, it seemed as though the future needed to be repressed, despite relatives mentioning attorney letters and insurances that needed handling. Nevertheless, participants did think about the future, and their thoughts were usually encumbered with fear of humiliation. Moreover, participants reported that they experienced their futures as being robbed from them and that it became insecure and unsafe because they did not know how the disease would progress and they knew they would not able to control it. Finally, their fear of changing their personalities to one that was different from the ones they had through their whole lives was experienced as humiliating and contributed to their fear of the future.

#### Fear of getting lost

The risk of forgetting where they were and the risk of getting lost caused fear for most participants. Stories in the news about disorientated people with dementia being tracked by police and helicopters were well known, and the thought of ending in a situation like this was expressed as the worst of possible humiliating situations. To prevent this, all participants went out of their homes with smartphones, so that they knew they could be monitored by the family through the GPS in the smartphone, which brought them safety—owing to knowing they were being monitored—and maintained peace of mind for both the participants and their families. They found it hard to understand the public debate about resistance to GPS-tracking for people with dementia. In that regard, a participant expressed:

“I cannot understand why I can’t just have a chip implanted. I could forget my smartphone. Animals can have a chip. If my family is okay with it—then I just really cannot understand why I can’t”. P4, man.

The participants reported that a GPS-tracker could give assurance at the sacrifice of their autonomy. The fear of the dementia progressing caused a risk of getting lost someday, and so the surveillance from GPS-tracking was preferred over the price of the possible humiliation.

#### Fear of getting a humiliating end-of-life

The fear of ending their life in a nursing home was also expressed as a fear of the future. The thought of being younger—than usual residents—and dependent on other people in a nursing home was difficult to face, since it is usually a place where very old people lived. The solution to avoid ending their life in nursing home had for some of the participants been to consider suicide. A participant narrated that he had savings for euthanasia in Switzerland as an alternative to a nursing home if the dementia disease became too deteriorating: “Life is always to prefer as related to die …, but how much is life worth to prefer …?” P5, man. He was of the opinion that death was preferred as related to live with young onset dementia when dementia progress and he risked losing sense of self and ending life at the nursing home. To him this was too humiliating.

This decision had caused different reactions in his family, but the fear of becoming severely ill from dementia, as he had seen in TV programs about people living with dementia, was too humiliating, and he preferred to end his life with dignity. References to TV programs about patients living with Alzheimer’s and the knowledge about dementia development—that was developed based on what they saw on the TV—clearly underlined that the future was associated with fear to all participants. The possibility of a lonely and humiliating future was something difficult for them to put into words, and is one of the facts that could explain why a voluntary ending of life was a choice for some of them.

## Discussion

The main findings in the current study underline that having young-onset dementia is experienced as losing control of oneself, becoming a burden to the family while the sense of self disappears, and fear of a humiliating future. This could be interpreted as a slow and painful loss of sense of self. The findings also provided new insights into their thoughts concerning surveillance at the expense of autonomy, and suicide as a way to obtain control and autonomy back to their lives.

The experience of losing sense of self can be understood as a nuanced feeling related to the struggles to live in life altered by the limitations of the diagnosis, which is also directly related to the experience of being a burden to others and forgetfulness, as Mazaheri et al. (2013) found in their study. We also believe that this loss of sense of self helps to illustrate the changes that the person with dementia witnesses, and how they cannot control them. The experience of losing sense of self is also consistent with Harris and Keady’s study on selfhood of younger people with dementia, where they also found there is a transition in selfhood and identity over various aspects of life because of loss of control (Harris & Keady, 2009).

The feeling of losing sense of self could also be explained by Gjødsbøl’s ethnographic research, which illustrates how the diagnosis and treatment of dementia challenge the fundamental values and principles of the autonomy of the people with dementia of being capable to make informed and rational choices about their own medical condition (Gjødsbøl & Svendsen, 2018). They found that, in the consultation, clinicians feel obliged to acknowledge the concerns articulated by relatives, and that the person with dementia is not necessarily a part of the conversation at counselling. Thus, if the person with dementia is perceived as a person who is not able to speak up for him/herself, the feeling of sense of self is at risk of being lost if the disease progresses.

Our results also showed that participants’ knowledge on the risk of becoming progressively unconscious in the future because of the disease contributed to the fear of losing sense of self. Further, the risk of not being recognized as the one you had been your whole life and the risk of being forgotten as the one you used to be contributed to losing sense of self. Martin Buber’s (1997) philosophy on I–Thou and I–It relationships can explain what it means to lose oneself when the relationship between the subject and how they are treated is reduced to I–It. An “I–it” is someone who is talked about as an object, an “I–Thou” is someone who is talked to as a person (Buber, 1997). Losing the ability to speak up for oneself can make the person with young onset dementia to feel like an “I–It” when they are no longer able to keep up with conversations; instead, they become someone who is still talked about in the room even though they are still present.

In a shift of the discussion over the results, the concerns related to being a burden to relatives when having dementia were also found in the previous research, and being burdensome has been shown to be of great concern to persons with dementia (Benbow & Kingston, 2016; Read, Toye, & Wynaden, 2017). Our results showed surprising findings related to the desire to prevent becoming a burden to close relatives, as some participants revealed their thoughts related to the consideration of suicide or that they would agree with “living in an open divorce”, as one participant expressed. A possible explanation of this could be participants’ fear of losing oneself that was reflected through their loss of control and loss of dignity, which were factors that an interpretative systematic review found were motivations for patients to wish to hasten death (Monforte-Royo, Villavicencio-Chávez, Tomás-Sábado, Mahtani-Chugani, & Balaguer, 2012). They found that a “wish to hasten death” was a way persons with dementia found to reduce their suffering related to being the one that was causing burden on the family, and also a way to relieve the burden of care from the family, so that they would not have to witness their progressive deterioration.

Dementia is an uncertain and unpredictable disease, and there are risks attached to its progression that challenge the person’s feeling of autonomy: the risk of feeling useless, of being a burden and of not being able to do anything unaided. A previous study points out, in a philosophical essay on autonomy and its competencies, that autonomy is the right to determine for oneself one’s own interests, goals and values, and one’s own conception of a good life, free from unwarranted interference (Atkins, 2006). In the current study, specific strategies such as considering suicide or living in open divorce could be ways that these persons with dementia find to determine their future for oneself in order to maintain control and autonomy over their lives.

The findings in our study also showed that persons with young onset dementia, in spite of their cognitive impairment and memory loss, are able to speak about their experiences of living with dementia, their coping strategies, their fears, their needs, and their wishes for the future. They were able to describe, in their own words, how it is to live with dementia, a fact that supported the research of Johannessen and Möller (2013).

The current study clearly showed that persons with young-onset dementia have their own opinion related to their situations and that they showed the desire to use GPS-tracking, while also not seeing any ethical issue in this process. They prefer giving up their privacy in exchange for living with the certainty that they will not experience the humiliation of getting lost. These results further support the findings of a previous study on how technology devices may be able to create a safe and secure environment for both the persons with dementia and their relatives (Olsson, Engström, Skovdahl, & Lampic, 2012). This possibility seems to overshadow the potential ethical problems, such as violating the integrity of the person with dementia. David Lyon suggests that surveillance has a dual nature; one side is used for protection, in a “caring” way, whilst the other side is used to regulate behaviour, in a “controlling” way (Lyon, 1995). When participants in our study reported they could not comprehend the reason behind not being able to choose whether they could be monitored or not, it demonstrated that there are intriguing questions underlying the nature and extent of the need for more surveillance, in a caring way, for people with dementia and its relationship with their feeling of safety and security.

Another important finding in the current study was how participants experienced the changes in their family relations, and how these changes caused feelings of fault and guilty conscience towards both their marriage and family members. In that regard, participants considered themselves as burdens towards their families, and it looked like there were issues too difficult to talk about within the family. Contrastingly, a previous research on how it is to be a family member to a person with dementia found the opposite; that family members experienced changed roles and new type dependency on each other within the family, which were also issues difficult to talk about within the family (Busted, Nielsen, & Birkelund, 2019). This newly changed roles and new types of dependency also resulted in a feeling of caregiver-burden, thereby leading to a need for support that should be provided to the whole family. The combination of the findings from the current study provided support for the conceptual premise that there is a noticeable need for involving the whole family when caring for persons with young-onset dementia.

### Methodological considerations

The interviews were conversational, so not all questions were posed in the same way to all participants, and while analysing this fact, it should also be taken into consideration that the setting and the participants' cognitive abilities were different. This could influence the results since the interviews were carried out very differently from one another. Nevertheless, the interviews were thorough and detailed, which helped to gain a wide understanding of the experiences of those living with young onset dementia.

A limitation of this study could be the small numbers of participants in the study. However, Malterud, Siersma, and Guassora (2016) identify different items having impact on the information power of the sample including narrow study aim, purposive, and specific sampling of participants, strong interview dialogue with thorough and rich descriptions of the experience of living with dementia and analysis strategy. This study is based on large information from thorough interview dialogues recruited from purposive and specific sampling of participants living with young onset dementia. The information power in this study was judged as sufficient even though the low number of participants. The analysis was supported by research and established theory which served to extend the sources of knowledge.

Reflexive thematic analysis was chosen to analyse data. This approach identifies patterns or themes within qualitative data. This analysis approach is not tied to a particular epistemological or theoretical perspective (Braun & Clarke, 2006). Given that the purpose of this study was to explore the experience of living with young onset dementia it was beneficial to use reflexive thematic analysis as a method. Another qualitative analysis method could have been chosen, e.g., grounded theory if the purpose had been to develop a specific theory. This study brings knowledge and theory on living with young onset dementia to enhance our understanding of the people living with this condition.

The family members’ presence during the interview could have both positive and negative impact on the results. It was a necessary step to meet these persons with dementia in the interview situation but it could be considered as a limitation to the study. In the cases with a family member being present during the interview, the participants showed, nonverbally, an obvious head-turning syndrome, and this could also help to explain losing sense of self. However, even people without dementia can have this action in conversations. The head-turning syndrome is found to be a clinical marker of Alzheimer’s disease and mild cognitive impairment and signifies the patients’ dependency on and trust in family members by shifting the responsibilities that the person with dementia is supposed to carry to those they trust (Fukui, Yamazaki, & Kinno, 2011). In this study, participants shown dependency on family members when losing the ability to find the right words in conversations or to finish sentences as usual, which could be a sign on creaking sense of self.

The interviewer's experience of working with people with dementia meant that this behaviour was expected. Knowing that interviewing persons with dementia calls for great patience and increased time, as the person with dementia may eventually hesitate for words and repeat narratives. Prolonged engagement and persistency during the interview required time and patience but made it possible to achieve a rich description on the experiences of those living with young onset dementia, which thereby ensured the validity of the interviews. The family members were briefed that they should avoid helping the patients when they were answering and were asked not to intervene or finish sentences for the participants which resulted in the participants to a large extent were willing to talk about their experiences, thoughts, and feelings. However, when the topic was sensitive, as an example about the participants’ thoughts about suicide the presence of the family members was a limitation to the dialogue and to the study. In two cases, it was clear that the participants protected their respective adult son/daughter during the interview situation, and so they hesitated to reveal their thoughts on suicide. As the participants started talking about the topic, just a little exposure already lead the family members to start crying, which clearly had an influence on which extent the participant explored and reported the topic.

Reflexive objectivity is defined as a reflection of one’s contribution as a researcher in the production of knowledge (Kvale & Brinkmann, 2015) and was required to increase reliability in the study. To be aware of the objective reflexivity, the first author responsible for the interviews and the first stages in the analysis process had to gain insight to own pre-understandings and being aware of when they appeared during both gathering data and analysing data (Attia & Edge, 2017). An openness and continuous awareness of pre-understandings involved all stages of the research process and were one way to increase reliability in this study (Kvale & Brinkmann, 2015).

### Conclusion

The experience of having and living with young onset dementia affects thoughts and memory and was experienced as losing sense of self. The feeling of losing control over mind and memory caused frustration and irritation towards oneself and was both a torment and an embarrassment in relation to other people. Further, we found that having young-onset dementia changed the family relations and caused the participants to feel at fault and with a guilty conscience towards both marriage and family when not being able to function as usual. These experiences resulted in the perception that they had become a burden to the family. Thoughts of the future were associated with fear, and the risk of changing their personalities to one that was different from that they had throughout most of their whole lives was experienced as humiliating. Participants’ fear of getting lost was experienced as the worst kind of humiliation possible, and underlined an increased need for surveillance, which should be carried out with care. It was also shown that persons with young-onset dementia preferred utilizing GPS-tracking, and did not see this type of usage as an ethical problem. Since we found reports of suicidal thoughts when being diagnosed with young-onset dementia, future studies are warranted to further investigate the topic to gather more knowledge in this regard.

### Implications for practice

The study has important implications for practice. It has been shown through our results that, when caring for persons with young onset dementia, there is a need to listen to their voices, in spite of their cognitive impairment and memory loss, since letting them speak about their experiences may assist them in regaining or maintaining a certain level of autonomy in their lives. These results are transferrable to other situations and settings where people with early-onset dementia live and act.

Further, there is a need for advanced care planning before the point at which cognition decreases critically is reached, so as to enlighten and empower persons with young-onset dementia to know their opportunities related to the influence they can have on their lives in the future even if diagnosed with dementia. This could contribute to the patients’ regaining control and autonomy over their lives and may perhaps help them in holding on to their sense of self.

Finally, future research should investigate how parents to persons with dementia experience their relations with their sons/daughters since this issue has yet to be given attention in academic research on the topic. This type of knowledge could greatly contribute to our understanding related to the involvement of the whole family when caring for persons with young-onset dementia. For this end, we believe that family health conversations may be one solution that allows for families that are dealing with the diagnosis to intervene early in the dementia illness process. This type of approach creates a context where all family members are able to narrate and reflect on each other’s stories, thereby increasing their understanding and manageability of the illness experience and its associated consequences (Benzein, Olin, & Persson, 2015). This type of approach could help to decrease the experience of strain and burden for the person with young onset dementia while also decreasing their feelings of fault and guilty conscience towards their own marriages and families.
